# Field-grown *ictB* tobacco transformants show no difference in photosynthetic efficiency for biomass relative to the wild type

**DOI:** 10.1093/jxb/erac193

**Published:** 2022-05-13

**Authors:** Ursula M Ruiz-Vera, Liana G Acevedo-Siaca, Kenny L Brown, Chidi Afamefule, Hussein Gherli, Andrew J Simkin, Stephen P Long, Tracy Lawson, Christine A Raines

**Affiliations:** Carl R. Woese Institute for Genomic Biology, University of Illinois at Urbana-Champaign, 1206 W Gregory Drive, Urbana, IL, USA; Bayer CropScience LLC, Bayer Marana Greenhouse, 9475 N Sanders Rd, Tucson, AZ, USA; Carl R. Woese Institute for Genomic Biology, University of Illinois at Urbana-Champaign, 1206 W Gregory Drive, Urbana, IL, USA; International Maize and Wheat Improvement Center (CIMMYT), México-Veracruz, El Batán Km. 45, Mexico; School of Life Sciences, University of Essex, Wivenhoe Park, Colchester CO4 3SQ, UK; N2 Applied AS, Hagaløkkveien 7, 1383 Asker, Norway; School of Life Sciences, University of Essex, Wivenhoe Park, Colchester CO4 3SQ, UK; School of Life Sciences, University of Essex, Wivenhoe Park, Colchester CO4 3SQ, UK; School of Biosciences, University of Kent, Canterbury CT2 7NJ, UK; Crop Science and Production Systems, NIAB-EMR, New Road, East Malling, Kent, UK; Carl R. Woese Institute for Genomic Biology, University of Illinois at Urbana-Champaign, 1206 W Gregory Drive, Urbana, IL, USA; Lancaster Environment Centre, University of Lancaster, Lancaster, UK; School of Life Sciences, University of Essex, Wivenhoe Park, Colchester CO4 3SQ, UK; School of Life Sciences, University of Essex, Wivenhoe Park, Colchester CO4 3SQ, UK; MPI of Molecular Plant Physiology, Germany

**Keywords:** Biomass production, crop production, *ictB* gene, photosynthesis, photosynthetic efficiency, water use efficiency

## Abstract

In this study, four tobacco transformants overexpressing the inorganic carbon transporter B gene (*ictB*) were screened for photosynthetic performance relative to the wild type (WT) in field-based conditions. The WT and transgenic tobacco plants were evaluated for photosynthetic performance to determine the maximum rate of carboxylation (*V*_c, max_), maximum rate of electron transport (*J*_max_), the photosynthetic compensation point (Γ*), quantum yield of PSII (Φ_PSII_), and mesophyll conductance (*g*_m_). Additionally, all plants were harvested to compare differences in above-ground biomass. Overall, transformants did not perform better than the WT on photosynthesis-, biomass-, and leaf composition-related traits. This is in contrast to previous studies that have suggested significant increases in photosynthesis and yield with the overexpression of *ictB*, although not widely evaluated under field conditions. These findings suggest that the benefit of ictB is not universal and may only be seen under certain growth conditions. While there is certainly still potential benefit to utilizing *ictB* in the future, further effort must be concentrated on understanding the underlying function of the gene and in which environmental conditions it offers the greatest benefit to crop performance. As it stands at present, it is possible that *ictB* overexpression may be largely favorable in controlled environments, such as greenhouses.

## Teaser

Despite previous studies showing potential for increased plant productivity through overexpression of the inorganic carbon transporter B gene (*ictB*), no significant difference was found between field-grown *ictB*-expressing tobacco lines and the wild type.

## Introduction

By the year 2050 it is projected that global food supply will need to increase by 50–85% to keep pace with a growing human population and shifting dietary preferences with greater emphasis on the consumption of animal products (Tilman *et al.*, 2011; Ray *et al*., 2012, 2013; Long *et al.*, 2015; FAO *et al*., 2020). As a result, yields of staple crops must increase at a considerably greater rate than today to ensure future food security. Furthermore, future crop varieties must be more sustainable and utilize water and nutrients more efficiently if they are to be environmentally sustainable (Foley *et al.*, 2011; Tilman *et al.*, 2011). While properties such as harvest index and light interception by the canopy have been improved close to their theoretical maxima over the past half century, little improvement has been made to photosynthetic efficiency in crop plants (Zhu *et al.*, 2008). Not only is the current rate of improvement in yield of crops plants insufficient to meet the projected future demand, but it may be stagnating (Long and Ort, 2010; Ray *et al.*, 2012; Long *et al.*, 2015). Increasing photosynthetic efficiency is a little exploited approach that holds great potential promise for improving yield and resource use efficiencies in crops (Zhu *et al.*, 2008; Long *et al.*, 2015).

Most major crops consumed by humans utilize the C_3_ photosynthetic pathway. C_3_ crops assimilate CO_2_ from the atmosphere inefficiently due to the lack of a carbon-concentrating mechanism, several internal resistances to CO_2_ diffusion, and because Rubisco is catalytically slow with a slow catalytic rate of CO_2_ assimilation in current atmospheric conditions (Tcherkez *et al.*, 2006; Price *et al.*, 2013; Erb and Zarzycki, 2018). The C_3_ photosynthetic process is also inefficient in its use of water and nitrogen (Parry *et al.*, 2011; Long *et al.*, 2018). Engineering a carbon-concentrating mechanism in C_3_ crops, much like those seen in C_4_ and cyanobacteria, would significantly reduce these inefficiencies (McGrath and Long, 2014; Long *et al.*, 2015). Indeed, many recent initiatives have aimed to improve C_3_ photosynthetic efficiency in crop plants to improve yield and productivity, such as the engineering of a C_4_ pathway in rice or constructing cyanobacterial carboxysomes in C_3_ chloroplasts (Mitchell and Sheehy, 2006; Long *et al.*, 2018; Ermakova *et al.*, 2020).

The inorganic carbon transporter B gene (*ictB*) is a highly conserved gene among cyanobacteria that was proposed to be involved in inorganic carbon accumulation in *Synechococcus* PCC 7942 (Bonfil *et al.*, 1998; Lieman-Hurwitz *et al.*, 2003; Price *et al.*, 2013). Previously, it was thought that *ictB* functioned as a carbon pump which could increase CO_2_ concentration within the leaf and improve photosynthesis (Lieman-Hurwitz *et al.*, 2003). Since then evidence has been presented showing that the *ictB* protein does not function as a HCO_3_^–^ transporter (Xu *et al.*, 2008; Price *et al.*, 2013), and therefore its function remains unknown (Simkin *et al.*, 2019).

Although the exact function of *ictB* is not yet known, several studies over the past 20 years have indicated that overexpressing *ictB* improves photosynthetic efficiency in C_3_ plants. ­Previously, Arabidopsis and tobacco transformants overexpressing *ictB* and grown in a controlled environment were found to have a significantly lower photosynthetic compensation point (Γ*) than the wild type (WT) (Lieman-Hurwitz *et al.*, 2003). This result suggested that increased *ictB* expression increased [CO_2_] at Rubisco, consequently increasing the carboxylation rate while competitively inhibiting oxygenation (Lieman-Hurwitz *et al.*, 2003; Hay *et al.* 2017). In greenhouse-grown tobacco, *ictB* expression led to an increase in the maximum rate of carboxylation (*V*_c, max_), the maximum rate of electron transport (*J*_max_), leaf CO_2_ uptake rate (*A*), and stomatal conductance (*g*_sw_) (Simkin *et al.*, 2015). Additionally, *ictB* expression may help boost photosynthetic performance in field conditions. Paddy-grown rice expressing *ictB* had significantly (10.5%) higher mesophyll conductance (*g*_m_) and 13.5% higher *A* compared with the WT (Gong *et al.*, 2015). Field-grown maize also benefited with increases in *A* and carbohydrate production, with increases in yield of up to 9.4% (Koester *et al.*, 2021). Replicated field trials of *ictB*-expressing soybean showed significant increases of 25% in *g*_m_, 14% in *A*, and 15% in seed yield relative to the WT (Hay *et al.*, 2017). Other studies have also noted increases in biomass production (Lieman-Hurwitz *et al.*, 2003; Yang *et al.*, 2008; Simkin *et al.*, 2015). Expression of *ictB* led to faster plant growth and greater accumulation of biomass under low-humidity conditions in Arabidopsis (Lieman-Hurwitz *et al.*, 2003) and higher overall biomass in soybean under water deprivation conditions (Hay *et al.*, 2017). Additionally, biomass increased by 71% in greenhouse-grown *ictB* tobacco transformants (Simkin *et al.*, 2015).

However, these gains may not always translate when grown in field conditions where improvements to crops would be most relevant towards improving food production. Indeed, previous studies have shown that *ictB* expression has not resulted in increased biomass (Gong *et al.*, 2015), except in drought conditions (Hay *et al.*, 2017). Previously, *ictB* tobacco transformants were shown to have increased photosynthetic performance and biomass without affecting water use efficiency (Simkin *et al.*, 2015). However, these transformants were only screened within the context of a controlled growth environment (Simkin *et al.*, 2015). In the present study, the tobacco transformants developed and utilized in Simkin *et al.* (2015) were grown in field conditions to evaluate their performance. The main objectives of this study were to (i) evaluate the photosynthetic performance of *ictB* mutants relative to the WT in field conditions, and (ii) assess the potential of *ictB* to improve water use efficiency in rain-fed field conditions. We subsequently discuss why benefits might be seen in greenhouses and controlled environments for *ictB* transformants but not in field trials.

## Materials and methods

### Growing conditions and germplasm

Tobacco transformants (ictB1, ictB3, ictB4, and ictB6) were produced at the University of Essex where the *ictB* single construct was placed in the tobacco (*Nicotiana tabacum*) cv. Samsun background (Simkin *et al.*, 2015). Tobacco transformants and WT tobacco plants were grown at the Energy Farm at the University of Illinois at Urbana-Champaign in Urbana, IL, USA. Seeds were sown into transplant trays on 9 July 2020, and transplanted into the field on 3 August 2020, in a random complete block design in which each genotype was replicated 12 times (Supplementary Fig. S1). Temperature (°C) and photosynthetic active radiation (PAR, µmol m^–2^ s^–1^) were measured throughout the field season (Supplementary Fig. S2). Once in the field, the plants were irrigated as needed to maintain soil moisture near field capacity (Supplementary Fig. S2). Measurements were made throughout August–September 2020. A full list of measured traits can be found in Supplementary Table S1.

### Gas exchange measurements

Leaf CO_2_ uptake and modulated chlorophyll fluorescence were measured on the youngest fully expanded leaves using portable open gas exchange systems incorporating CO_2_ and water vapor infra-red gas analyzers (LI-6800, LI-COR Biosciences, Lincoln, NE, USA). Light was provided through an integrated LED light source and modulated fluorometer, incorporated into the head of the temperature- and humidity-controlled leaf measurement chamber (6 cm^2^, LI-6800-01A, LI-COR Biosciences).

### CO_ 2_ and light response curves

The response of CO_2_ uptake (*A*, µmol CO_2_ m^–2^ s^–1^) to intercellular CO_2_ concentration (*C*_i_, µmol mol^–1^) and to photosynthetic photon flux density (PPFD) was measured twice during the experiment. Response curves were performed 47 d after sowing (from 24 to 27 August 2020) and once again later in development at 61 d after sowing (from 7 to 10 September 2020). Response curves were measured for each genotype once per each block (*n*=12).

To examine the response of *A* to *C*_i_ (*A*/*C*_i_ curves), photosynthesis was measured at saturating light (2000 µmol m^–2^ s^–1^) and CO_2_ concentrations in the following order: 400, 250, 150, 100, 50, 400, 550, 700, 900, 1100, 1300, and 1500 µmol mol^–1^. Additionally, the block temperature was set at 28 °C, the average relative humidity was between 66% and 77%, and the vapor pressure deficit (VPD) at leaf temperature was between 0.79 kPa and 1.74 kPa. The gas exchange systems were matched before each curve, and steady-state fluorescence (*F*_s_) and maximal light-adapted fluorescence (*F*_m_ʹ) were recorded at each measured *C*_i_.

The apparent *V*_c, max_ (µmol m^–2^ s^–1^) and apparent *J*_max_ (µmol m^–2^ s^–1^) were calculated utilizing the equations from von Caemmerer and Farquhar (1981). Due to the changes in ambient temperature throughout the day, the leaf temperature was variable (raw data in Supplementary Fig. S3). Accordingly, the temperature response curves from Bernacchi et al. (2001, 2003) were applied to obtain the apparent *V*_c, max_ and apparent *J*_max_ at 28 °C. The ‘apparent’ term is used because the parameters are based on *C*_i_ instead of CO_2_ concentration inside the chloroplast (*C*_c_). The photorespiratory CO_2_ compensation point (Γ*, µmol mol^–1^), carboxylation efficiency (CE, µmol m^–2^ s^–1^ µbar^–1^), and the maximum rate of CO_2_ uptake in saturating light and CO_2_ (*A*_max_, µmol m^–2^ s^–1^) were calculated from the *A*/*C*_i_ curves that were fitted at 28 °C. CE was the initial slope of curves with *C*_i_ ≤250 µmol mol^–1^.

A non-linear analysis with the Marquardt method (Moualeu-Ngangue *et al.*, 2017) that uses the equations from the variable *J* method to calculate *g*_m_ (mol m^–2^ s^–1^) (Harley *et al.*, 1992) and equations from von Caemmerer and Farquhar (1981) and Farquhar and von Caemmerer (1982) were then used to obtain *C*_c_ (µmol mol^–1^), *V*_c, max_, and *J*_max_. For this analysis, the scaling constant (*c*) and the enthalpies of activation (Δ*H*_a_) to calculate the Michaelis constant of Rubisco for CO_2_ (*K*_c_; µmol mol^–1^), the inhibition constant (*K*_o_; µmol mol^–1^), and Γ* at 25 °C were taken from Sharkey *et al.* (2007). Then, the *V*_c, max_, *J*_max_, and *g*_m_ were adjusted to 28 °C using the equations in Bernacchi et al. (2001, 2002, 2003).

The Γ* adjusted (Γ*_adjusted) for *g*_m_ was calculated as in Furbank *et al.* (2009) and Walker and Cousins (2013): Γ*_adjusted=Γ*+*R*_d_/*g*_m_, where *R*_d_ is the daytime respiration rate obtained from the *A*/*C*_*i*_ curves.

Light response curves (*A/Q* curves) were measured at ambient [CO_2_] (400 µmol mol^–1^) and the following PPFDs: 2000, 1700, 1400, 1100, 800, 600, 425, 250, 150, 100, 50, and 0 µmol m^–2^ s^–1^. The gas exchange systems were matched before each curve, and *F*_s_ and *F*_m_ʹ were recorded at each PPFD. The *A/Q* curves were fitted for quantum efficiency (Φ_PSII_), leaf CO_2_ uptake in saturated light (*A*_sat_, µmol m^–2^ s^–1^), and light compensation point utilizing the {photosynthesis} R-package (R Core Team, 2020; Stinziano *et al.*, 2021), which uses the Marshall and Biscoe (1980) non-rectangular hyperbola model.

### Diurnal measurements

Diurnal measurements were made every 2 h on 3 September 2020, from 08.00 h to 18.00 h. On this day, sunrise was at ~06.23 h, while sunset was at ~19.20 h. One plant of each genotype was measured in each of the 12 blocks per time point (*n*=12/genotype/time point). Within the cuvette, the flow rate was 500 µmol s^–1^, [CO_2_] was maintained at 400 µmol mol^–1^, relative humidity was maintained at 70%, and actinic PPFD was 10% blue light. The PPFD and block temperature were changed at each time point to reflect ambient conditions throughout the day. The gas exchange systems were matched before each time point measurement, and *F*_s_ and *F*_m_ʹ were logged. The parameters of *A*, stomatal conductance (*g*_sw_, mol H_2_O m^−2^ s^−1^), *C*_i_, and intrinsic water use efficiency (iWUE=*A*/*g*_sw_, µmol CO_2_ mol H_2_O^−1^) throughout the course of a day were obtained from these data.

### Confirmation of *ictB* expression

Leaf discs were collected into liquid N_2_ the day following the diurnal measurements (4 September 2020) from one plant per tobacco genotype per block (*n*=12 per genotype). After the samples were ground, total RNA and protein were extracted from the same leaf discs using the NucleoSpin RNA/Protein Kit (Macherey-Nagel, http://www.mn-net.com). Once the protocol was completed, the RNA concentration was diluted to 200 ng μl^–1^.

cDNA was synthesized using 1 μg of total RNA in 20 μl using the oligo(dT) primer (Invitrogene) according to the protocol in the RevertAid Reverse Transcriptase kit (Fermentas, Life Sciences, UK). The cDNA was diluted 10 times. For semi-quantitative reverse transcription–PCR (RT–PCR), 10 μl of cDNA in a total volume of 25 μl was used with HS VeriFi Mix (PCR Biosystems Ltd., UK) according to the manufacturer’s recommendations. The PCR products were fractionated on 2.0% agarose gels. qPCRs were prepared with the 2× qPCRBIO SyGreen Mix Lo-ROX (PCR Biosystems Ltd., UK) with 1 µl of cDNA and 0.5 µM of each primer in a total volume of 10 µl. The amplification reaction included 40 cycles of 5 s at 95 °C, 10 s at 60 °C, and 15 s at 72 °C. The expression level of *ictB* was normalized with the values obtained for the housekeeping gene for protein phosphatase 2A (PP2A; Supplementary Fig. S4). Primers in 5ʹ–3ʹ orientation used were RT-PCR-ictB-Fw, AGCCAAACTGACGCTCTACC; RT-PCR-ictB-Rv,CGCGACTGTAGGTGAGGATC; qPCR-ictB-Fw, GTTGGTTTTTGCCCTAGCGG; qPCR-ictB-Rv, TTGGTTGAGGCCGTAGACAC; qPCR-PP2A-Fw, GTGAAGCTGTAGGGCCTGAGC; and qPCR-PP2A-Rv, CATAGGCAGGCACCAAATCC.

### Determination of leaf carbon and leaf carbon isotopic composition

Leaf discs were collected on 4 September 2020. Samples were freeze-dried and ground. Then, ~2 mg of each leaf sample was used to ­determine the carbon content (leaf C, %) and the carbon isotopic composition (δ^13^C, ‰) using an elemental analyzer (Costech 4010, Costech Analytical Technologies, Valencia, CA, USA) in conjunction with an isotope ratio mass spectrometer (DeltaV Advantage, Thermo Fisher Scientific, Bremen, Germany) on continuous flow. The carbon ratios were then measured relative to laboratory standards and calibrated relative to the international Vienna Pee Dee Belemnite (VPDB) standard.

### Destructive harvest and biomass quantification

All tobacco plants (~48 plants per genotype) were harvested on 16 September 2020 to obtain the total number of leaves, number of leaves on the main stem, total leaf area (cm^2^), and stem height (cm) per plant. Total leaf area was measured using a leaf area meter (LI-3100C Area Meter, LI-COR Environmental, Lincoln, NE, USA). Biomass samples were dried to a constant weight at 50 °C to determine leaf dry and stem dry weight (g per plant). The above-ground biomass was the combined sum of leaf and stem dry weight. Leaf area ratio (LAR, cm^2^ g^–1^) was determined by dividing the total leaf area by the total above-ground biomass.

### Statistical analyses

After testing for normal distribution, homogeneity of variances by the Shapiro–Wilk test and Levine test, variables were analyzed with a mixed model ANOVA with or without repeated measurements. ‘Day’ was the repeated measurement factor when a variable was collected multiple times throughout the season. The fixed effects were the genotype (tobacco lines), day, and their interactions, while the block was the random effect. The Kenward–Roger method was used to calculate the degrees of freedom. Mean discrimination analysis was performed utilizing Tukey’s honest significant difference (HSD) with significance determined as *P*-value ≤0.05. Statistical analyses and model fitting for the *A/Q* curves and diurnal measurements were performed in R (version 4.01, R-Project). The rest of the analyses were done in SAS (version 9.4, SAS Institute Inc., Cary, NC, USA), by using the PROC UNIVARIATE procedure to assess for normality and for the discovery of outliers, and by using the PRO MIXED procedure for the ANOVA. Pair-wise comparisons were done by the least square means test (*t*-test) with significance determined as a *P*-value ≤0.05.

## Results

### Confirmation of *ictB* expression in transgenic plants

The ictB transgenic lines used in this study are the same as those presented in Simkin *et al.* (2015). Semi-quantitative RT–PCR was used to detect the presence of the transcript in the *ictB*-expressing plant lines ictB1, ictB3, ictB4, and ictB6. No transcript was detected in WT control plants, and different levels of transgene expression were observed among transgenic lines, with ictB6 showing the highest transgene expression (Supplementary Fig. 6A). qPCR was performed to validate the differences in transgene expression between lines. No signal was detected in WT plants and ictB6 showed the highest transgene expression (Supplementary Fig. 6B). Both results are consistent and indicate that the *ictB* transgene is expressed in transgenic lines at different levels, and these results are also consistent with the data presented in Simkin *et al.* (2015).

### Gas exchange data: CO_2_ response curves, light response curves, and diurnals

**Fig. 1.**
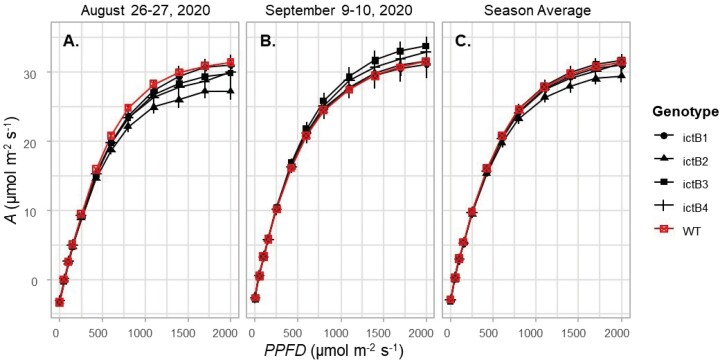
CO_2_ uptake (*A*) response to change in light (PPFD) in *ictB* tobacco transformants (ictB1, ictB3, ictB4, and ictB6) and wild-type (WT) tobacco. The light response curves were measured in ambient [CO_2_] conditions (~400 mol mol^–1^). Each point is the mean (±SE) of 12 plants.

*A/Q* curves were measured to allow for the determination of parameters related to how efficiently the plant is utilizing light. *A*/*Q* curves were measured on 12 plants per line (*n*=12). No significant differences were found between genotypes for *A*_sat_, Φ_PSII_, and light compensation point for any of the *A*/*Q* curve measurements throughout the season (Fig. 1). While not significant, the WT had one of the highest photosynthetic rates in the first set of *A/Q* curves but not in the second set (Fig. 1). However, indicated differences were small (Fig. 1).

**Fig. 2.**
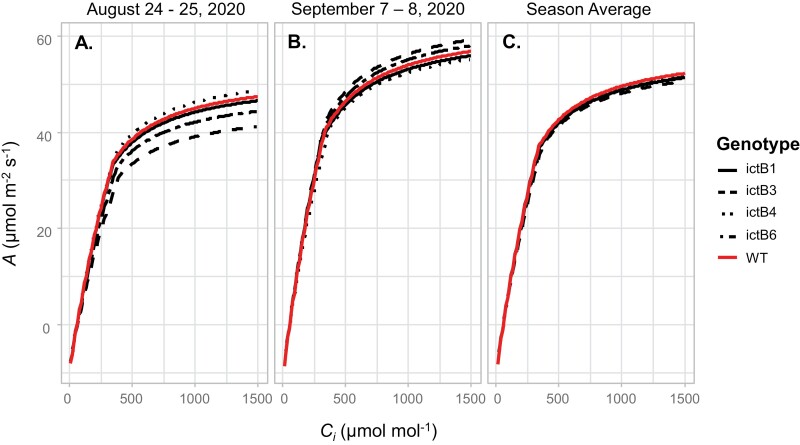
CO_2_ uptake (*A*) response to change in intercellular CO_2_ concentration (*C*_i_) fitted at 28 °C in *ictB* tobacco transformants (ictB1, ictB3, ictB4, and ictB6) and wild type (WT) tobacco. The CO_2_ response curves were measured in saturating light conditions (2000 µmol m^–2^ s^–1^). Raw data are provided in Supplementary Fig.S2.

**Fig. 3.**
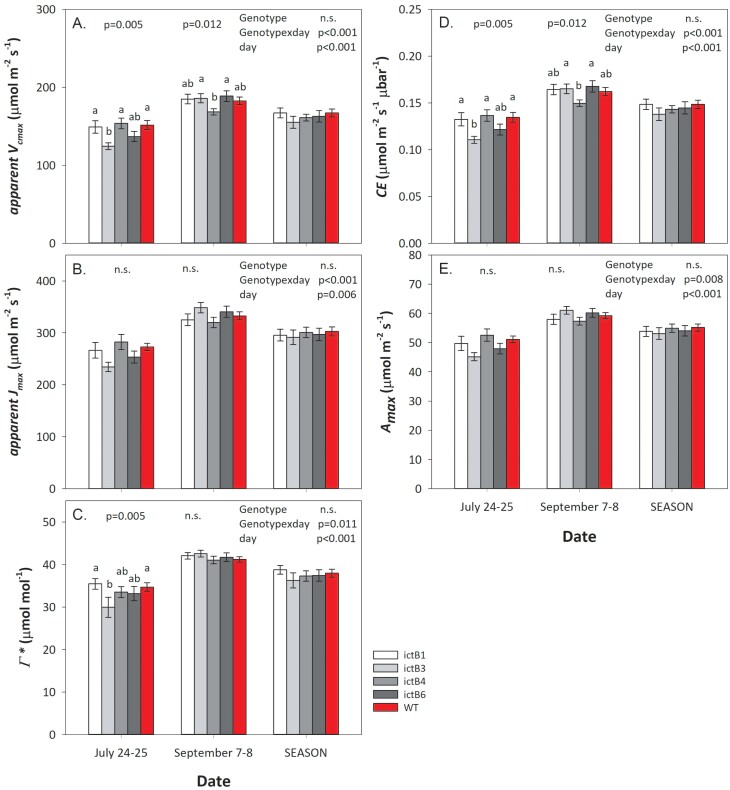
The ‘apparent’ maximum rate of carboxylation (apparent *V*_c, max_), the ‘apparent’ maximum rate of electron transport (apparent *J*_max_), the compensation point (Γ*), carboxylation efficiency (CE), and the maximum rate of CO_2_ uptake in saturating light and CO_2_ (*A*_max_) based on *A/C*_i_ curves at 28 °C for *ictB* tobacco transformants and wild-type (WT) tobacco. Each point is the mean (±SE) of 8–12 plants per genotype. Results of the complete block analysis of variance (ANOVA) for the season and for each day of measurements are at the top of each panel. Pair-wise comparisons (*t*-test) are indicated with letters on top of the bars; transformants with different letters represent statistically significant differences (*P*<0.05).

The *A/C*_i_ curves were measured to determine parameters related to the biochemical performance and limitation of photosynthesis. These were also measured on 12 plants per line (*n*=12). The apparent *V*_c, max_, apparent *J*_max_, CE, *A*_max_, and Γ* in the transgenic lines were not significantly higher than in the WT. The overall values of these parameters increased throughout the duration of the season, but without significant differences between lines (Figs 2, 3). Exceptions were that ictB3 had a lower apparent *V*_c, max_ and CE than the WT, ictB1, and ictB4 during the first set of measurements (Fig. 3). ictB3 had also a lower Γ* than the WT and ictB1 at the beginning of the season (Fig. 3). By the end of the field season, ictB4 had an apparent *V*_c, max_ and CE that were lower than in ictB3 and ictB6 (Fig. 3). When considering the parameters calculated based on *C*_c_, *V*_c, max_, *J*_max_, and Γ*_adjusted did not differ between the transgenic lines and the WT (Supplementary Fig. S5). ictB3 was the only transgenic with a *g*_m_ lower than the WT, although the difference was only significant on one date (Supplementary Fig. S5).

**Fig. 4.**
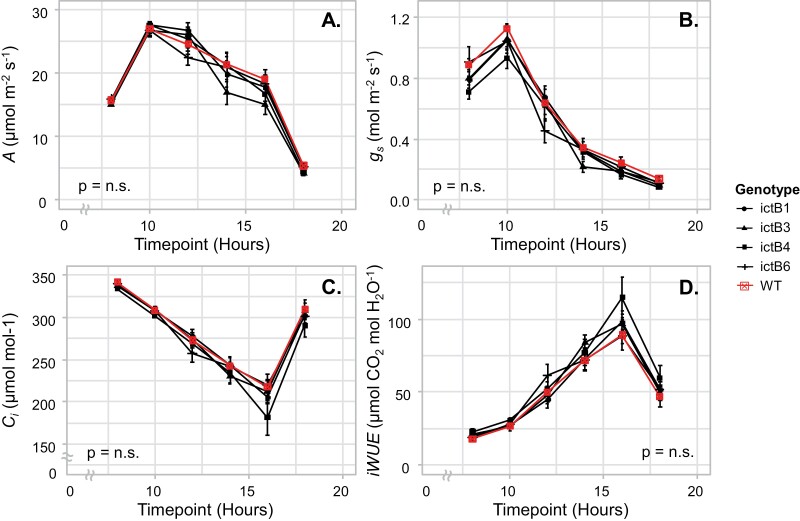
Photosynthetic parameters CO_2_ uptake (*A*), stomatal conductance (*g*_sw_), intrinsic water-use efficiency (iWUE=*A*/*g*_s_), and intercellular CO_2_ concentration (*C*_i_) measured in diurnal measurement in five genotypes, four of which (ictB1, ictB3, ictB4, and ictB6) being transgenic transformants expressing inorganic carbon transporter B (ictB). Diurnal measurements were made every 2 h on 3 September 2020, from 08.00 h through 18.00 h. On this day, sunrise was at ~06.23 h, while sunset was at ~19.20 h. Each point is the mean (±SE) of 12 plants. ANOVA results are at the bottom of each panel, with significance determined as a *P*-value ≤0.05. The ≈ symbol denotes an axis break.

Finally, no significant differences were found between the genotypes for *A*, *g*_sw_, *C*_i_, and iWUE during the diurnal gas exchange measurement (Fig. 4). While the WT had the lowest overall iWUE, it was not significantly lower in the transgenic lines (Fig. 4).

### Leaf composition and biomass-related traits

**Fig. 5.**
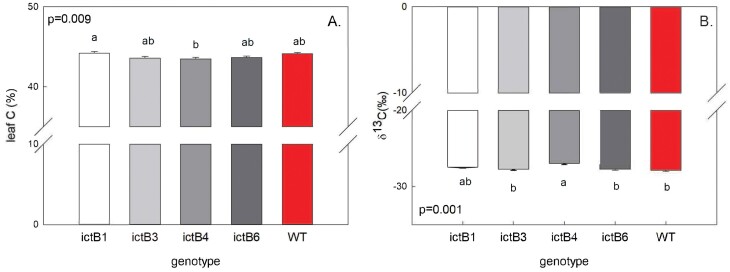
Leaf carbon content and leaf carbon isotope composition (δ^13^C). Each bar is the mean (±SE) of ~12 samples. Results of the complete block ANOVA for the season and for each day of measurements are at the top of each panel. Pair-wise comparisons (*t*-test) are indicated with letters on top of the bars; transformants with different letters represent statistically significant differences (*P*<0.05).

Leaf carbon content (leaf C) and δ^13^C varied significantly among the measured genotypes (Fig. 5). None of the transformants showed a leaf C content that was significantly different from that of the WT; however, ictB1 showed a significantly higher content than ictB4 (Fig. 5). The WT had the lowest value (most negative) for δ^13^C although it only varied significantly from the ictB4 genotype (Fig. 5). The δ^13^C values from all the ictB genotypes were compared (mean value of –27.48‰) against the δ^13^C in the WT (mean value of –27.88‰), showing a significantly more negative δ^13^C in the WT (*P*-value=0.040).

**Fig. 6.**
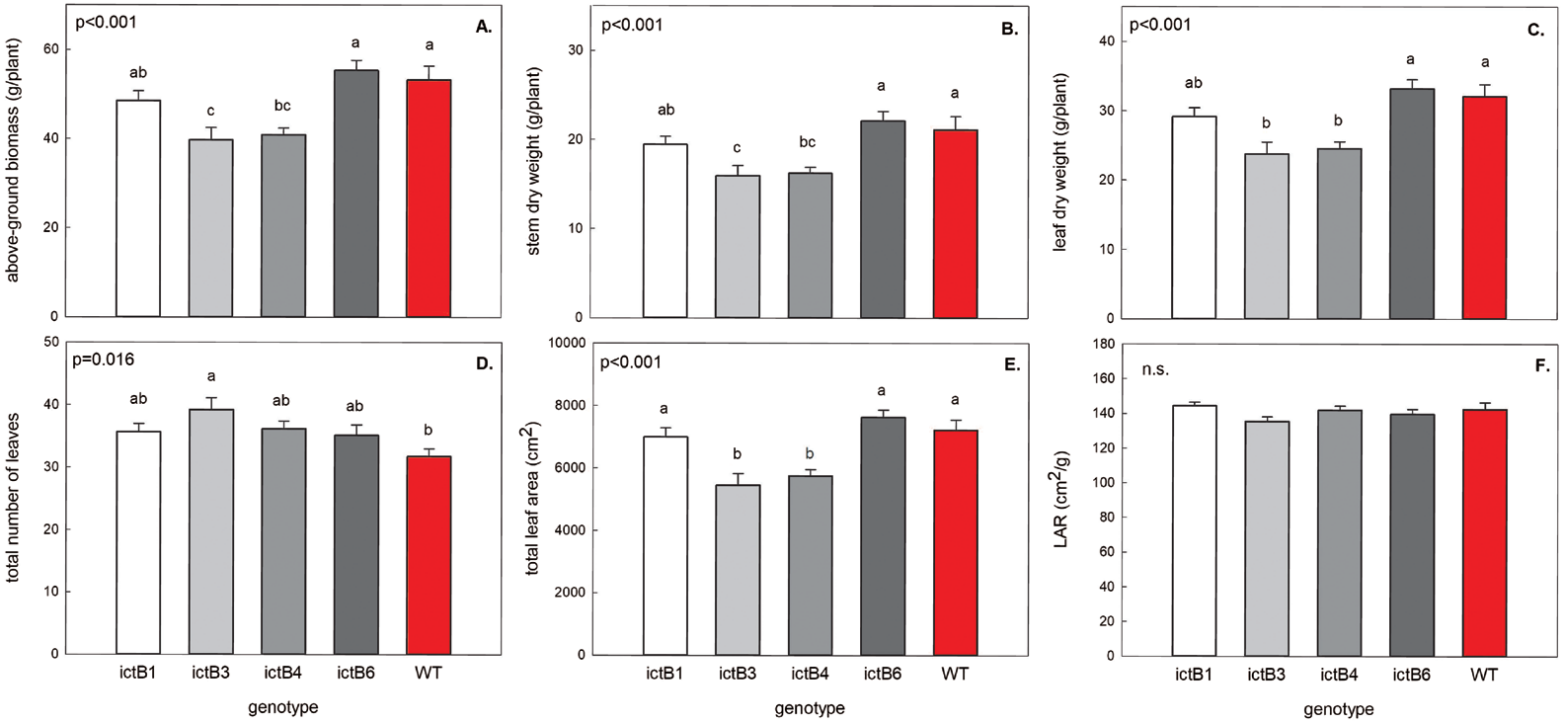
Biomass data collected from destructive harvest of the five genotypes measured. Each bar is the mean (±SE) of ~48 plants. Results of the complete block ANOVA for the season and for each day of measurements are at the top of each panel. Pair-wise comparisons (*t*-test) are indicated with letters on top of the bars; transformants with different letters represent statistically significant differences (*P*<0.05).

Significant differences were found among the genotypes for most measured biomass-related traits, including above-ground biomass, leaf and stem dry weights, total number of leaves, number of leaves on the main stem, total leaf area, and stem height (Fig. 6; Supplementary Fig. S6). Despite having the lowest total number of leaves, the WT had one of the highest total above-ground biomasses, total leaf area, and leaf dry weights (Fig. 6; Supplementary Fig. S6). The WT had significantly lower total number of leaves and number of leaves on the main stem than the ictB3 transformant. The WT also had higher above-ground biomass, stem dry weight, leaf dry weight, total leaf area, and stem height than both ictB3 and ictB4 transformants (Fig. 6; Supplementary Fig. S6). Finally, the pair-wise comparisons for LAR did not reveal significant differences between the lines (Fig. 6).

## Discussion

Previous reports of plants transformed with the *ictB* gene indicated higher photosynthesis and biomass compared with the WT plants from which they were derived (Lieman-Hurwitz *et al*., 2003, 2005; Simkin *et al* 2015; Hay *et al* 2017). However, most of these studies have been performed in controlled conditions and it is not clear if these promising improvements in plant productivity can translate to the crops in the field. For this reason, in this experiment we grew *ictB* tobacco plants in the field to evaluate if these transgenic plants have a higher photosynthetic efficiency than the WT under field conditions. A total of four *ictB* transgenic lines were tested against tobacco WT plants from which they were derived, and were evaluated for >10 different photosynthetic parameters together with leaf composition and biomass traits (Supplementary Table S1).

The same transgenic lines were used previously in the greenhouse study of Simkin *et al.* (2015). In that experiment, overall higher photosynthesis, apparent *V*_c, max_, apparent *J*_max_, and *g*_sw_ were found in these *ictB* lines, resulting in more leaves and stem biomass. In this experiment, we did not find any photosynthetic parameter that was higher in *ictB* tobacco compared with the WT (Figs 1–4; Supplementary Fig. S5). In contrast, *ictB* tobacco performed similarly to the WT, although one transgenic line (ictB3) had a lower apparent *V*_c, max_, CE, and *g*_m_ than the WT in at least one of the sets of measurements (Fig. 3; Supplementary Fig. S5). The lower *g*_m_ in ictB3 indicated a higher restriction to the diffusion of CO_2_ inside the chloroplast than in the WT. However, ictB3 did show a lower Γ*, which suggests an increased concentration of CO_2_ around Rubisco. However, when Γ* was adjusted to consider the effect of *g*_m_, Γ*_adjusted did not indicate a higher amount of CO_2_ around Rubisco in ictB3 or in any other *ictB* line compared with the WT (Supplementary Fig. S5). Previous studies of plants transformed with *ictB* have calculated Γ* from *A/C*_i_ response curves, without accounting for *g*_m_ (Lieman-Hurwitz *et al.*, 2003; Gong *et al.*, 2015; Hay *et al.*, 2017). The present study indicates the importance of calculating Γ* based on *C*_c_ instead of *C*_i_ for studies where the calculation of this parameter can allow a better understanding of any photosynthetic improvement achieved.

The values of apparent *V*_c, max_ and apparent *J*_max_ from this study were also obtained at 25 °C (Supplementary Table S2) to compare them with the values obtained in Simkin *et al.* (2015) which were calculated at that temperature. In our field experiment, the apparent *V*_c, max_ at 25 °C was between 95 µmol m^–2^ s^–1^ and 145 µmol m^–2^ s^–1^, while the apparent *J*_max_ was between 195 µmol m^–2^ s^–1^ and 290 µmol m^–2^ s^–1^, considering both *ictB* lines and the WT. These values are higher than the apparent *V*_c, max_ (between 70 µmol m^–2^ s^–1^ and 90 µmol m^–2^ s^–1^) and the apparent *J*_max_ (between 130 µmol m^–2^ s^–1^ and 170 µmol m^–2^ s^–1^) obtained in the greenhouse study of Simkin *et al.* (2015). It is possible that under the controlled growth conditions of the greenhouse, differences could be apparent that were later eliminated in the field. Similarly, in an *ictB* soybean study (Hay *et al.*, 2017), the apparent *V*_c, max_ and apparent *J*_max_ were not different from the WT when grown in the field under ambient CO_2_ concentrations; however, soybean instantaneous photosynthesis and biomass did increase. It is important to note that soybean as a legume can have an adequate nitrogen supply throughout the whole growing season, which might have contributed to its increase in carbon assimilation. In Ruiz-Vera *et al.* (2017), WT tobacco cv. Petit Havana, another tobacco cultivar with reduced sink capacity due to its determinate growth, grew at normal and high N soil fertilization conditions in the field. In that study, the values of apparent *V*_c, max_ and apparent *J*_max_ increased further under the high N treatment at ambient CO_2_ conditions. It might be that under field conditions, other factors such as adequate nutrient supply and uptake or sink strength can influence the effect of the *ictB* gene in tobacco. Our results suggest that it is possible to maximize the photosynthetic performance of *ictB* plants over the WT under conditions where parameters such as the amount of light, temperature, relative humidity, photoperiod, nutrients, and water availability can be controlled (e.g. conditions in the *ictB* tobacco greenhouse experiment in Simkin *et al.*, 2015), but this improvement might not always translate to field conditions.

The values for *A* and other parameters obtained from the *A/C*_i_ and *A/Q* curves were higher at the end of the season compared with the beginning of the season (Figs 1–3; Supplementary Fig. S3). This trend corresponds to previous work in which values related to photosynthesis increase with leaf age (Bielczynski *et al.*, 2017). This difference could have been influenced by the hotter temperatures during the days when the first set of measurements were carried out (approximately +5 °C; Supplementary Fig. S2) which could have also increased the water requirements of the plants. Previous studies suggest that *ictB* plants might have higher water use efficiency (WUE) than the WT because *A* and *g*_m_ tend to be higher while *g*_sw_ does not change (Lieman-Hurwitz *et al.*, 2003; Gong *et al.*, 2015; Hay *et al.*, 2017). In our experiment, iWUE was not significantly different between *ictB* tobacco and the WT (Fig. 4), which coincided with the lack of significant differences seen, in most of the cases, for *A*, *g*_*s*w_, *g*_m_, and δ^13^C. However, ictB4 did show significantly less negative δ^13^C than the WT, and a less negative δ^13^C was indicated for ictB1 (Fig. 5). The δ^13^C of leaf tissue provides an integrated signal of the WUE with which the carbon in that leaf was obtained. A less negative value indicates a higher WUE, provided that *g*_m_ is not different between lines. These results suggest that the transformation with *ictB* may improve WUE under field conditions, although the scope may be limited (Fig. 5).

The results regarding the effect of ictB on crop biomass production have so far been inconclusive. For example, in some cases, *ictB*-overexpressing plants have produced significantly greater biomass (Lieman-Hurwitz *et al.*, 2003; Hay *et al.*, 2017; Koester *et al.*, 2021), while in other studies overexpression of *ictB* did not significantly alter biomass production (Gong *et al.*, 2015). This suggests that the mechanism that underlies ictB may be greatly affected by environmental factors, and that an increase in crop productivity may only happen under certain conditions, although these conditions have yet to be identified. Here, there were no increases in biomass in *ictB* tobacco; on the contrary, some *ictB* lines (ictB3 and ictB4) had lower biomass (above-ground biomass, leaf biomass, and stem biomass; Fig. 6) than the WT, probably because of the production of smaller leaves (total leaf area; Fig. 5) and shorter plants (Supplementary Fig. S6). This study did not measure root biomass; however, empirical observations in the previous greenhouse experiment indicated that *ictB* lines might have more root biomass than the WT (Simkin *et al.*, 2015). Despite that, the effect of the *ictB* expression in plants remains unclear (Simkin *et al.*, 2019); its effect might be enhanced when it is co-expressed with other genes such as with some Calvin–Benson cycle genes (Simkin *et al.*, 2017). For example, higher dry biomass was observed in plants with the *ictB* gene together with the overexpression of sedoheptulose-1,7-bisphosphatase (SBPase) and fructose-1,6-bisphosphate aldolase (FBPase) (Simkin *et al.*, 2017). Consequently, the impact of *ictB* on the improvement of photosynthesis and yield may be observed when it is part of a group of expressed genes rather than when it is expressed alone.

### Future applications for *ictB*-overexpressing plants

Despite not finding a significant difference between the *ictB* transformants and the WT except possibly in WUE, this study was done in one field season so the replication of our results in multiple seasons is unknown. Moreover, there is still promise in utilizing expression of this gene for improved crop productivity, particularly in controlled environments. While we did not find the significant differences in biomass that were reported in Simkin *et al.* (2015), it is possible that field environmental conditions, which differ greatly from potted plants in the constant conditions of greenhouses and growth cabinets, play a key role in whether plants transformed with *ictB* perform better relative to their WT. This, combined with the successes seen in greenhouse-grown *ictB* transformants, could serve as encouragement for deploying this gene to improve plant productivity within the context of controlled environments. Indeed, as humans globally look to increase food production in more sustainable ways, greater emphasis has been placed on agriculture in greenhouses and vertical farming, both of which involve controlling the growing environment.

Other considerations that could be taken into account are the water status of the plant, the temperature, and the age of the plant, as all of these are factors that can play a role in how a transgene manifests itself in the field and affects photosynthetic performance (Azcon-Bieto *et al*., 1981). At present, it is difficult to know the true potential of transformation of crop plants with *ictB*, especially as the function of the gene remains unknown (Simkin *et al.*, 2019).

Drought and heat conditions adversely affect photosynthetic performance in crops, including increasing the photorespiratory CO_2_ compensation point and decreasing Rubisco carboxylation or ribulose bisphosphate (RuBP) regeneration (Antolin and Sanchez-Diaz, 1993; Rensburg and Kruger, 1993; Flexas *et al.*, 2009; Crous *et al.*, 2013). Consequently, increases in temperature are associated with an increase in photorespiration in C_3_ plants, which can lead to yield penalties of up to 36% in important food crops (Walker *et al.*, 2016). Previously, it was shown that expression of *ictB* in plants resulted in a decrease of Γ* (Lieman-Hurwitz *et al.*, 2003; Hay *et al.* 2017). Testing *ictB* transformants under conditions that are known to affect the CO_2_ compensation point could help us to better understand the underlying function and see if improved performance is significantly associated with specific environmental factors. For example, if *ictB* transformants can maintain a lower Γ* under drought or heat stress conditions, then it could be a promising application for the future, especially as temperature and drought stress are projected to increase with global climate change. However, as mentioned, further effort would need to be put into understanding under which specific conditions *ictB* might be most beneficial.

## Supplementary data

The following supplementary data are available at *JXB* online.

Fig. S1. Layout of the field experiment.

Fig. S2. Weather data for the growing season.

Fig. S3. Raw data for *A*/*C*_i_ curves.

Fig. S4. Semi-quantitative RT–PCR and qPCR results of transgenic lines.

Fig. S5. Comparison of *V*_c, max_, *J*_max_, *g*_m_, and Γ*_adjusted between transgenic lines and the wild type at 28 °C.

Fig. S6. Stem height and number of leaves on the main stem.

Table S1. Summary of traits measured, their abbreviations, and units.

Table S2. Apparent *V*_c, max_ and *J*_max_ at 25 °C .

erac193_suppl_Supplementary_Material

## Data Availability

Data are available upon request via the corresponding author.

## References

[CIT0001] Antolín MC , Sánchez-Díaz M. 1993. Effects of temporary droughts on photosynthesis of alfalfa plants. Journal of Experimental Botany 44, 1341–1349.

[CIT0002] Azcón-Bieto J , Farquhar GD, Caballero A. 1981. Effects of temperature, oxygen concentration, leaf age and seasonal variations on the CO_2_ compensation point of *Lolium perenne* L. Planta 152, 497–504. 24301153 10.1007/BF00380820

[CIT0003] Bernacchi CJ , Pimentel C, Long SP. 2003. *In vivo* temperature response functions of parameters required to model RuBP-limited photosynthesis. Plant, Cell & Environment 26, 1419–1430.

[CIT0004] Bernacchi CJ , Portis AR , Nakano H , von Caemmerer S , Long SP. 2002. Temperature response of mesophyll conductance. Implications for the determination of Rubisco enzyme kinetics and for limitations to photosynthesis *in vivo*. Plant Physiology 130, 1992–1998. 12481082 10.1104/pp.008250 PMC166710

[CIT0005] Bernacchi CJ , Singsaas EL, Pimentel C, Portis ARJ, Long SP. 2001. Improved temperature response functions for models of Rubisco-limited photosynthesis. Plant, Cell & Environment 24, 253–259.

[CIT0006] Bielczynski LW , Lacki MK, Hoefnagels I, Gambin A, Croce R. 2017. Leaf and plant age affects photosynthetic performance and photoprotective capacity. Plant Physiology 175, 1634–1648. 29018097 10.1104/pp.17.00904 PMC5717728

[CIT0007] Bonfil DJ , Ronen-Tarazi M, Sültemeyer D, Lieman-Hurwitz J, Schatz D, Kaplan A. 1998. A putative HCO_3_^–^ transporter in the cyanobacterium *Synechococcus* sp. strain PCC 7942. FEBS Letters 430, 236–240. 9688546 10.1016/s0014-5793(98)00662-0

[CIT0008] Crous KY , Quentin AG, Lin YS, Medlyn BE, Williams DG, Barton CVM, Ellsworth D. 2013. Photosynthesis of temperate *Eucalyptus globulus* trees outside their native range has limited adjustment to elevated CO_2_ and climate warming. Global Change Biology 19, 3790–3807. 23824839 10.1111/gcb.12314

[CIT0009] Erb TJ , Zarzycki J. 2018. A short history of RubisCO: the rise and fall (?) of Nature’s predominant CO_2_ fixing enzyme. Current Opinion in Biotechnology 49, 100–107. 28843191 10.1016/j.copbio.2017.07.017 PMC7610757

[CIT0010] Ermakova M , Danila FR, Furbank RT, von Caemmerer S. 2020. On the road to C_4_ rice: advances and perspectives. The Plant Journal 101, 940–950. 31596523 10.1111/tpj.14562 PMC7065233

[CIT0011] FAO, IFAD, UNICEF, WFP, WHO. 2020. The State of Food Security and Nutrition in the World 2020. Transforming food systems for affordable healthy diets. Rome, FAO. doi:10.4060/ca9692en.

[CIT0012] Farquhar GD , von Caemmerer S. 1982. Modelling of photosynthetic response to environmental conditions. In: Lange OL, Nobel PS, Osmond CB, Ziegler H, eds. Physiological plant ecology II. Berlin, Heidelberg: Springer, 549–587.

[CIT0013] Flexas J , Barón M, Bota J, et al. 2009. Photosynthesis limitations during water stress acclimation and recovery in the drought-adapted *Vitis* hybrid Richter-110 (*V. berlandieri×V. rupestris*). Journal of Experimental Botany 60, 2361–2377. 19351904 10.1093/jxb/erp069

[CIT0014] Foley JA , Ramankutty N, Brauman KA, et al. 2011. Solutions for a cultivated planet. Nature 478, 337–342. 21993620 10.1038/nature10452

[CIT0015] Furbank RT , von Caemmerer S, Sheehy J, Edwards GE. 2009. C_4_ rice: a challenge for plant phenomics. Functional Plant Biology 36, 845–859. 32688695 10.1071/FP09185

[CIT0016] Gong HY , Li Y, Fang G, Hu DH, Jin WB, Wang ZH, Li YS. 2015. Transgenic rice expressing *Ictb* and *FBP/Sbpase* derived from cyanobacteria exhibits enhanced photosynthesis and mesophyll conductance to CO_2_. PLoS One 10, e0140928. doi:10.1371/journal.pone.0140928. 26488581 PMC4638112

[CIT0017] Harley PC , Loreto F, Di Marco G, Sharkey TD. 1992. Theoretical considerations when estimating the mesophyll conductance to CO_2_ flux by analysis of the response of photosynthesis to CO_2_. Plant Physiology 98, 1429–1436. 16668811 10.1104/pp.98.4.1429 PMC1080368

[CIT0018] Hay WT , Bihmidine S, Mutlu N, Hoang KL, Awada T, Weeks DP, Clemente TE, Long SP. 2017. Enhancing soybean photosynthetic CO_2_ assimilation using a cyanobacterial membrane protein, ictB. Journal of Plant Physiology 212, 58–68. 28273517 10.1016/j.jplph.2017.02.003

[CIT0019] Koester RP , Pignon CP, Kesler DC, et al. 2021. Transgenic insertion of the cyanobacterial membrane protein ictB increases grain yield in *Zea mays* through increased photosynthesis and carbohydrate production. PLoS One 16, e0246359. 33539477 10.1371/journal.pone.0246359 PMC7861388

[CIT0020] Lieman-Hurwitz J , Asipov L, Rachmilevitch S, Marcus Y, Kaplan A. 2005. Expression of cyanobacterial ictB in higher plants enhanced photosynthesis and growth. In: Omasa K, Nouchi I, De Kok LJ, eds. Plant responses to air pollution and global change. Tokyo: Springer, 133–138.

[CIT0021] Lieman-Hurwitz J , Rachmilevitch S, Mittler R, Marcus Y, Kaplan A. 2003. Enhanced photosynthesis and growth of transgenic plants that express *ictB*, a gene involved in HCO_3_^–^ accumulation in cyanobacteria. Plant Biotechnology Journal 1, 43–50. 17147679 10.1046/j.1467-7652.2003.00003.x

[CIT0022] Long BM , Hee WY, Sharwood RE, et al. 2018. Carboxysome encapsulation of the CO_2_-fixing enzyme Rubisco in tobacco chloroplasts. Nature Communications 9, 3570. 10.1038/s41467-018-06044-0 PMC6120970 30177711

[CIT0023] Long SP , Ort DR. 2010. More than taking the heat: crops and global change. Current Opinion in Plant Biology 13, 241–248. 20494611 10.1016/j.pbi.2010.04.008

[CIT0024] Long SP , Marshall-Colon A, Zhu XG. 2015. Meeting the global food demand of the future by engineering crop photosynthesis and yield potential. Cell 161, 56–66. 25815985 10.1016/j.cell.2015.03.019

[CIT0025] McGrath JM , Long SP. 2014. Can the cyanobacterial carbon-concentrating mechanism increase photosynthesis in crop species? A theoretical analysis. Plant Physiology 164, 2247–2261. 24550242 10.1104/pp.113.232611 PMC3982776

[CIT0026] Mitchell PL , Sheehy JE. 2006. Supercharging rice photosynthesis to increase yield. New Phytologist 171, 688–693. 16918541 10.1111/j.1469-8137.2006.01855.x

[CIT0027] Moualeu-Ngangue DP , Chen T-W, Stützel H. 2017. A new method to estimate photosynthetic parameters through net assimilation rate–intercellular space CO_2_ concentration (*A*–*C*_*i*_) curve and chlorophyll fluorescence measurements. New Phytologist 213, 1543–1554. 27768807 10.1111/nph.14260

[CIT0028] Parry MA , Reynolds M, Salvucci ME, Raines C, Andralojc J, Zhu XG, Price GD, Condon AG, Furbank RT. 2011. Raising yield potential of wheat. II. Increasing photosynthetic capacity and efficiency. Journal of Experimental Botany 62, 453–467. 21030385 10.1093/jxb/erq304

[CIT0029] Price GD , Pengelly JJL, Forster B, Du J, Whitney SM, von Caemmerer S, Badger MR, Howitt SM, Evans JR. 2013. The cyanobacterial CCM as a source of genes for improving photosynthetic CO_2_ fixation in crop species. Journal of Experimental Botany 64, 753–768. 23028015 10.1093/jxb/ers257

[CIT0030] R Core Team. 2020. R: a language and environment for statistical computing. Vienna, Austria: R Foundation for Statistical Computing.

[CIT0031] Ray DK , Mueller ND, West PC, Foley JA. 2013. Yield trends are insufficient to double global crop production by 2050. PLoS One 8, e66428. 23840465 10.1371/journal.pone.0066428 PMC3686737

[CIT0032] Ray DK , Ramankutty N, Mueller ND, West PC, Foley JA. 2012. Recent patterns of crop yield growth and stagnation. Nature Communications 3, 1293. 10.1038/ncomms2296 23250423

[CIT0033] Rensburg LV , Krüger GHJ. 1993. Comparative analysis of differential drought stress-induced suppression of and recovery in carbon dioxide fixation: stomatal and non-stomatal limitation in *Nicotiana tabacum* L. Journal of Plant Physiology 142, 296–306.

[CIT0034] Ruiz-Vera UM , De Souza AP, Long SP, Ort DR. 2017. The role of sink strength and nitrogen availability in the down-regulation of photosynthetic capacity in field-grown *Nicotiana tabacum* L. at elevated CO_2_ concentration. Frontiers in Plant Science 8, 998. doi:10.3389/fpls.2017.00998. 28649261 PMC5465258

[CIT0035] Sharkey TD , Bernacchi CJ, Farquhar GD, Singsaas EL. 2007. Fitting photosynthetic carbon dioxide response curves for C_3_ leaves. Plant, Cell & Environment 30, 1035–1040. 10.1111/j.1365-3040.2007.01710.x 17661745

[CIT0036] Simkin AJ , Lopez-Calcagno PE, Davey PA, Headland LR, Lawson T, Timm S, Bauwe H, Raines CA. 2017. Simultaneous stimulation of sedoheptulose 1,7-bisphosphatase, fructose 1,6-bisphophate aldolase and the photorespiratory glycine decarboxylase h-protein increases CO_2_ assimilation, vegetative biomass and seed yield in Arabidopsis. Plant Biotechnology Journal 15, 805–816. 27936496 10.1111/pbi.12676 PMC5466442

[CIT0037] Simkin AJ , Lopez-Calcagno PE, Raines CA. 2019. Feeding the world: improving photosynthetic efficiency for sustainable crop production. Journal of Experimental Botany 70, 1119–1140. 30772919 10.1093/jxb/ery445 PMC6395887

[CIT0038] Simkin AJ , McAusland L, Headland LR, Lawson T, Raines CA. 2015. Multigene manipulation of photosynthetic carbon assimilation increases CO_2_ fixation and biomass yield in tobacco. Journal of Experimental Botany 66, 4075–4090. 25956882 10.1093/jxb/erv204 PMC4473996

[CIT0039] Stinziano JR , Roback C, Sargent D, Murphy BK, Hudson PJ, Muir CD. 2021. Principles of resilient coding for plant ecophysiologists. AoB Plants 13, plab059. 34646435 10.1093/aobpla/plab059 PMC8501907

[CIT0040] Tcherkez GGB , Farquhar GD, Andrews TJ. 2006. Despite slow catalysis and confused substrate specificity, all ribulose bisphosphate carboxylases may be nearly perfectly optimized. Proceedings of the National Academy of Sciences, USA 103, 7246–7251. 10.1073/pnas.0600605103 PMC1464328 16641091

[CIT0041] Tilman D , Balzer C, Hill J, Befort BL. 2011. Global food demand and the sustainable intensification of agriculture. Proceedings of the National Academy of Sciences, USA 108, 20260–20264. 10.1073/pnas.1116437108 PMC3250154 22106295

[CIT0042] von Caemmerer S , Farquhar GD. 1981. Some relationships between the biochemistry of photosynthesis and the gas exchange of leaves. Planta 153, 376–387. 24276943 10.1007/BF00384257

[CIT0043] Walker BJ , VanLoocke A, Bernacchi CJ, Ort DR. 2016. The costs of photorespiration to food production now and in the future. Annual Review of Plant Biology 67, 107–129. 10.1146/annurev-arplant-043015-111709 26865340

[CIT0044] Walker BJ , Cousins AB. 2013. Influence of temperature on measurements of the CO_2_ compensation point: differences between the Laisk and O_2_-exchange methods. Journal of Experimental Botany 64, 1893–1905. 23630324 10.1093/jxb/ert058 PMC3638825

[CIT0045] Xu M , Bernát G, Singh A, Mi H, Rögner M, Pakrasi HB, Ogawa T. 2008. Properties of mutants of *Synechocystis* sp. strain PCC 6803 lacking inorganic carbon sequestration systems. Plant & Cell Physiology 49, 1672–1677. 18784196 10.1093/pcp/pcn139

[CIT0046] Yang S-M , Chang C-Y, Yanagisawa M, Park I, Tseng T-H, Ku MSB. 2008. Transgenic rice expressing cyanobacterial bicarbonate transporter exhibited enhanced photosynthesis, growth and grain yield. In: Allen JF, Gantt E, Golbeck J, Osmond B, eds. Photosynthesis. Energy from the Sun. Dodrecht: Springer, 1243–1246. doi:10.1007/978-1-4020-6709-9_268.

[CIT0047] Zhu XG , Long SP, Ort DR. 2008. What is the maximum efficiency with which photosynthesis can convert solar energy into biomass? Current Opinion in Biotechnology 2, 153–159. 10.1016/j.copbio.2008.02.004 18374559

