# Supplementary material for: Field-grown ictB tobacco transformants show no difference in photosynthetic efficiency for biomass relative to the wild type
Source: J Exp Bot. 2022 May 13;73(14):4897–907. doi: 10.1093/jxb/erac193 (PMC9366323; doi:10.1093/jxb/erac193)
Supplement: erac193_suppl_Supplementary_Material [file erac193_suppl_supplementary_material.pdf]

Supplementary Information:

Supplementary Figure S1. Layout of field trial of ictB tobacco transformants at the Energy Farm at the University of Illinois at Champaign-Urbana. Tobacco plants were transplanted in a random complete block design with 12 blocks. The azygous transformant (AZY) data was not used in this study.

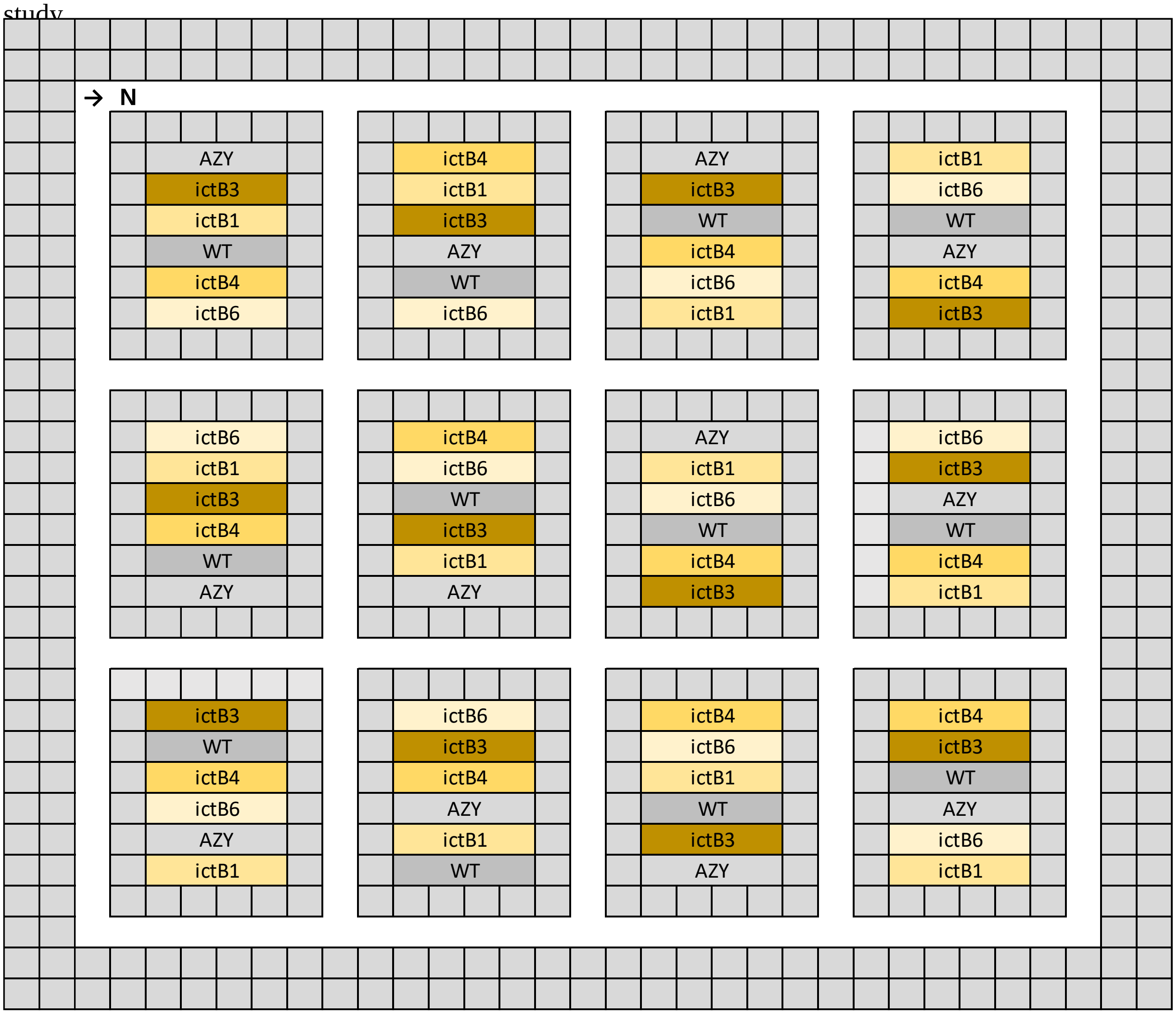

Supplementary Figure S2. A. Mean, minimum and maximum temperature and **B.** Maximum photosynthetic active radiation for each day of the tobacco growing season. The days when irrigation was done are indicated with a cross symbol, the days when there was rainfall are indicated with triangles. Transplanting day is indicated with red and the days of measurements with white circles.

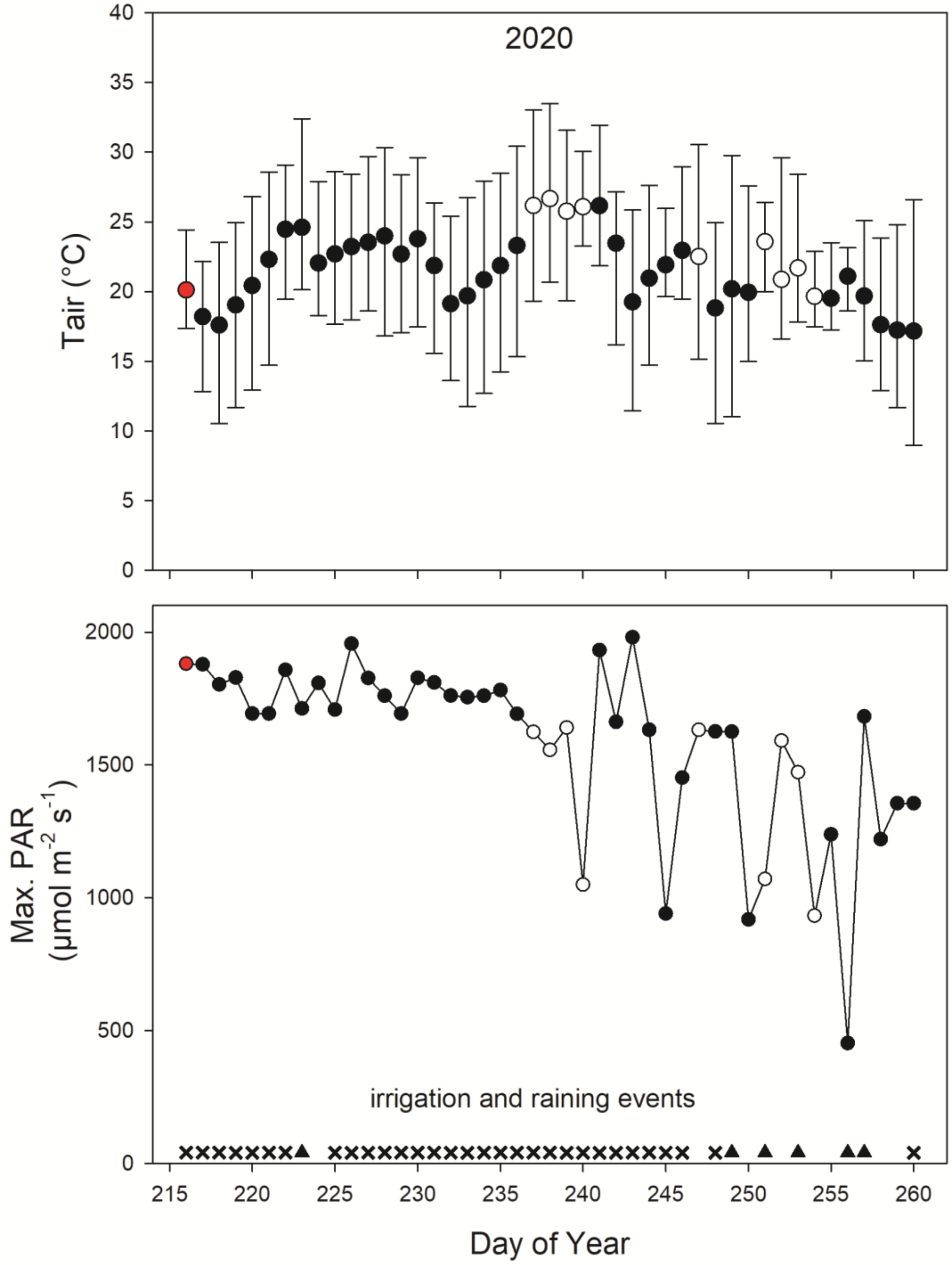

Supplementary Figure S3. Raw data of CO_2 uptake (*A*) response to change in intercellular CO_2 concentration (C_i) at saturated light (2000 µmol m^{-2} s^{-1}) in *ictB* tobacco transformants (ictB1, ictB3, ictB4, ictB6) and wildtype (WT) tobacco. Leaf temperature varied between 29.0°C to 34.0°C in August and varied between 26.3°C to 31.9°C in September. Between eight to twelve curves per genotype were used for the A/C_i curve analysis.

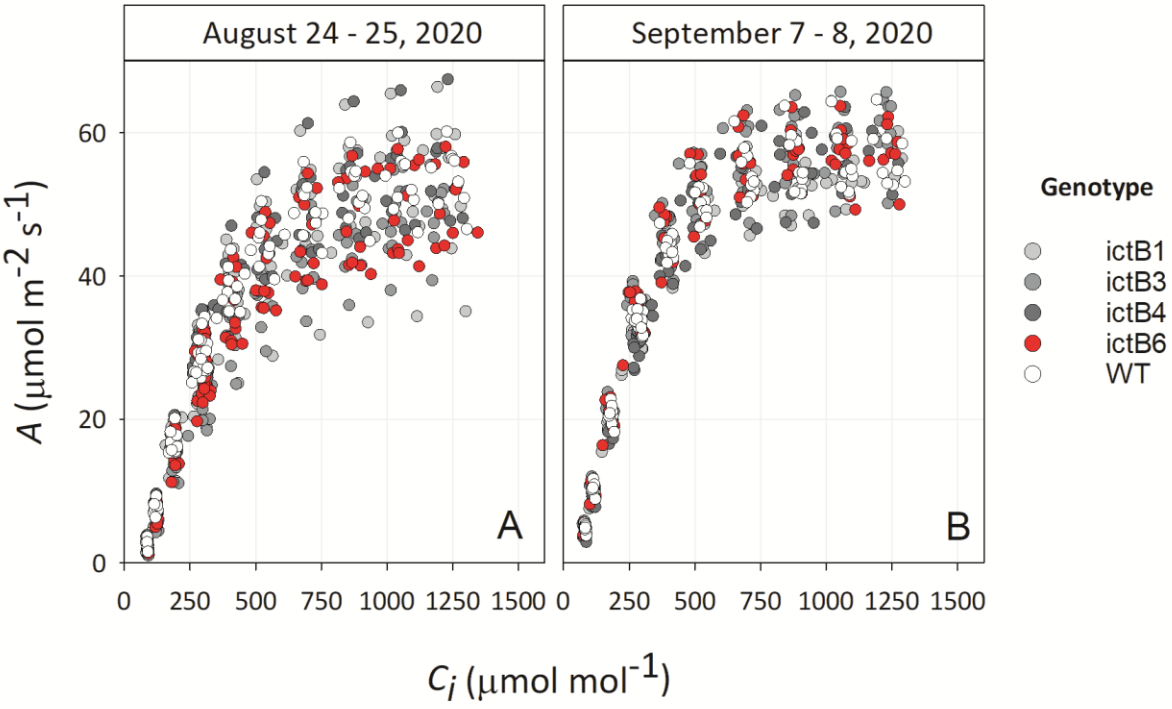

Supplementary Figure S4. Semi-quantitative RT-PCR and qPCR results of transgenic lines. A. ictB transcript levels in WT and transgenic lines. B. ictB expression level relative to endogenous gene PP2A in transgenic lines. Error bars are SD.

A

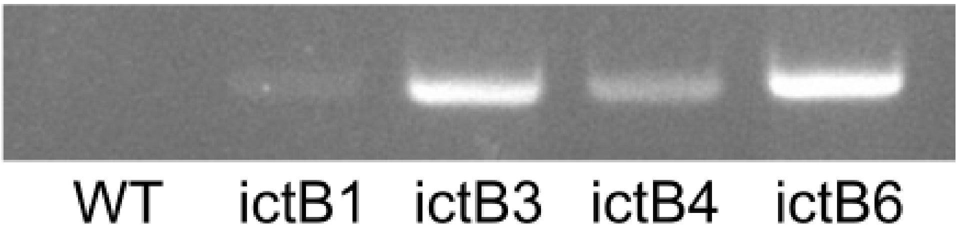

B

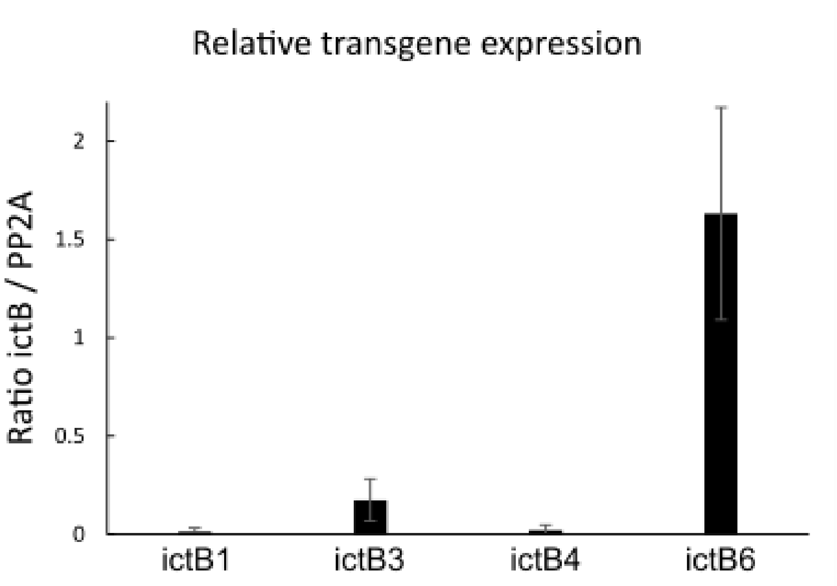

Supplementary Figure S5. A. The maximum rate of carboxylation ($V_{c,\,max}$), **B.** the maximum rate of electron transport (J_{max}), **C.** mesophyll conductance at 28º C, and **D.** the adjusted photosynthetic compensation point (Γ*_*adjusted*) for *ictB* tobacco transformants and wildtype (WT) tobacco. Each point is the mean (± SE) of eight to twelve plants per genotype. Results of the complete block analysis of variance (ANOVA) for the season and for each day of measurements are at the top of each panel. Pair-wise comparisons (*t*-test) are indicated with letters on top of the bars; transformants with different letters represent statistically significant differences ($p < 0.05$).

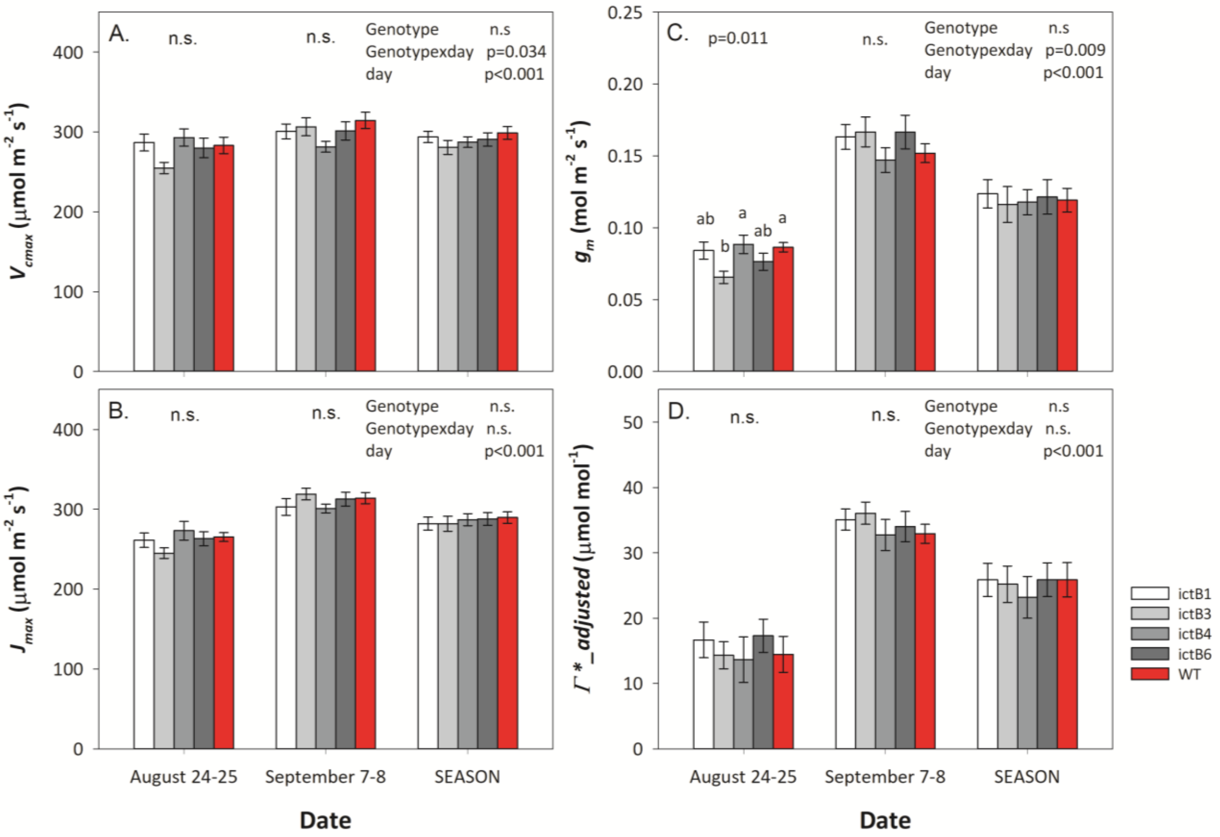

Supplementary Figure S6. Stem height and number of leaves on the main stem. Data was collected from destructive harvest of the five genotypes measured. Each bar is the mean (± SE) of ~48 plants. Results of the complete block analysis of variance (ANOVA) are at the top of each panel. Pair-wise comparisons (*t*-test) are indicated with letters on top of the bars; transformants with different letters represent statistically significant differences ($p < 0.05$).

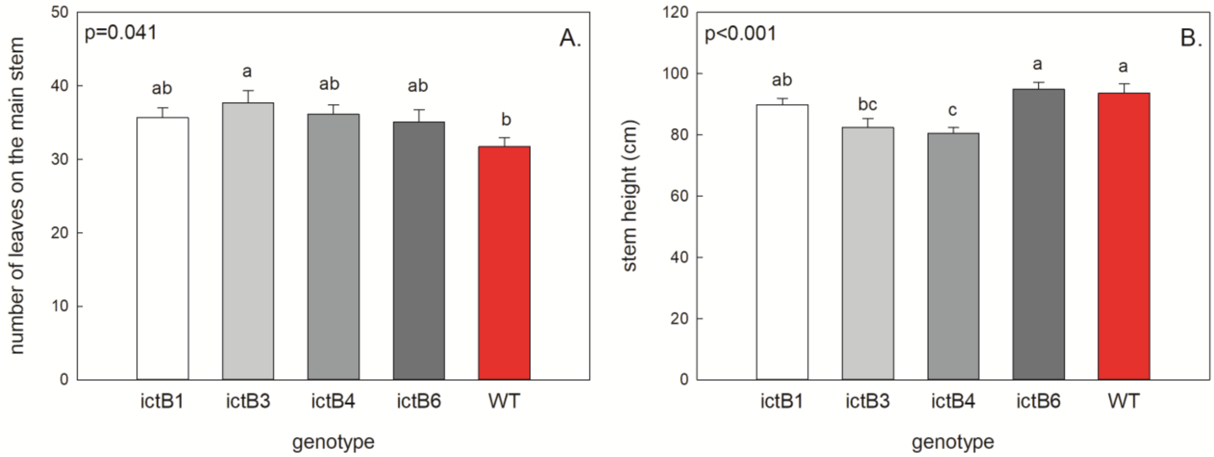

Supplementary Table S1. A summary of traits measured and their units.

Trait	Description	Unit
A	CO_2 uptake	µmol m^{-2} s^{-1}
g_{sw}	Stomatal conductance of water	mol m^{-2} s^{-1}
C_i	Intercellular CO_2 concentration	µmol mol^{-1}
C_c	CO_2 concentration inside the chloroplast	µmol mol^{-1}
iWUE	Intrinsic water-use efficiency (iWUE = A/g_{sw})	µmol CO_2 mol H_2O^{-1}
Φ_{PSII}	Quantum yield of PSII	Unitless (0-1)
apparent $V_{c,\,max}$	Maximum rate or carboxylation based on A/C_i curves	µmol m^{-2} s^{-1}
apparent J_{max}	Maximum rate of electron transport based on A/C_i curves	µmol m^{-2} s^{-1}
CE	Carboxylation efficiency	µmol m^{-2} s^{-1} µbar^{-1}
Γ^*	CO_2 compensation point based on A/C_i curves	µmol mol^{-1}
A_{max}	CO_2 uptake in saturating [CO_2] and light	µmol m^{-2} s^{-1}
$V_{c,\,max}$	Maximum rate or carboxylation based on A/C_c curves	µmol m^{-2} s^{-1}
J_{max}	Maximum rate of electron transport based on A/C_c curves	µmol m^{-2} s^{-1}
g_m	Mesophyll conductance	mol m^{-2} s^{-1}
Γ^**_adjusted*	CO_2 compensation point considering g_m	µmol mol^{-1}
A_{sat}	CO_2 uptake in saturated light	µmol m^{-2} s^{-1}
Leaf C	Leaf carbon content	%
$\delta^{13}C$	Leaf carbon isotopic composition	‰
Above-ground biomass	Total biomass, leaves and stems	g $plant^{-1}$
Total number of leaves	Total number of leaves at harvest, on main stem and branches	leaves
Number of leaves on the main stem	Number of leaves only on the main stem, at harvest	leaves
Total leaf area	Fresh leaf area	cm^2
Stem height	Stem length at harvest	cm
Leaf dry weight	Leaf dry weight	g $plant^{-1}$
Stem dry weight	Stem dry weight	g $plant^{-1}$
LAR	Leaf area ratio	cm^2 g^{-1}

Supplementary Table S2. The "apparent" maximum rate of carboxylation (*apparent* $V_{c, max}$), the "apparent" maximum rate of electron transport (*apparent* J_{max}) based on A/C_i curves at 25º C for *ictB* tobacco transformants and wildtype (WT) tobacco. The *apparent* $V_{c, max}$ and *apparent* J_{max} values were obtained from eight to twelve curves per genotype.

	Tobacco transformants	*apparent* *Vcmax* at 25°C	± SE	*apparent* *Jmax* at 25°C	± SE
24-25 August	ictB1	115.3	6.16	223.3	12.68
	ictB3	96.3	3.30	196.6	7.54
	ictB4	118.9	5.32	236.8	12.21
	ictB6	105.8	5.03	212.4	9.79
	WT	117.3	4.46	228.9	5.90
7-8 September	ictB1	143.0	4.82	272.6	9.35
	ictB3	143.8	4.50	292.1	8.36
	ictB4	130.3	2.99	268.2	8.23
	ictB6	145.9	5.24	285.5	8.99
	WT	141.2	3.73	278.9	6.60
